# Antibody-Mediated Autoimmune Diseases of the CNS: Challenges and Approaches to Diagnosis and Management

**DOI:** 10.3389/fneur.2021.673339

**Published:** 2021-07-07

**Authors:** Elia Sechi, Eoin P. Flanagan

**Affiliations:** ^1^Department of Neurology, Mayo Clinic, Rochester, MN, United States; ^2^Department of Medical, Surgical and Experimental Sciences, University of Sassari, Sassari, Italy; ^3^Department of Laboratory Medicine and Pathology, Mayo Clinic, Rochester, MN, United States

**Keywords:** limbic encephalitis/encephalopathy, immune checkpoint inhibitors, autoantibody testing, paraneoplastic, myelin oligodendrocyte glycoprotein, aquaporin-4

## Abstract

Antibody-mediated disorders of the central nervous system (CNS) are increasingly recognized as neurologic disorders that can be severe and even life-threatening but with the potential for reversibility with appropriate treatment. The expanding spectrum of newly identified autoantibodies targeting glial or neuronal (neural) antigens and associated clinical syndromes (ranging from autoimmune encephalitis to CNS demyelination) has increased diagnostic precision, and allowed critical reinterpretation of non-specific neurological syndromes historically associated with systemic disorders (e.g., Hashimoto encephalopathy). The intracellular vs. cell-surface or synaptic location of the different neural autoantibody targets often helps to predict the clinical characteristics, potential cancer association, and treatment response of the associated syndromes. In particular, autoantibodies targeting intracellular antigens (traditionally termed onconeural autoantibodies) are often associated with cancers, rarely respond well to immunosuppression and have a poor outcome, although exceptions exist. Detection of neural autoantibodies with accurate laboratory assays in patients with compatible clinical-MRI phenotypes allows a definite diagnosis of antibody-mediated CNS disorders, with important therapeutic and prognostic implications. Antibody-mediated CNS disorders are rare, and reliable autoantibody identification is highly dependent on the technique used for detection and pre-test probability. As a consequence, indiscriminate neural autoantibody testing among patients with more common neurologic disorders (e.g., epilepsy, dementia) will necessarily increase the risk of false positivity, so that recognition of high-risk clinical-MRI phenotypes is crucial. A number of emerging clinical settings have recently been recognized to favor development of CNS autoimmunity. These include antibody-mediated CNS disorders following herpes simplex virus encephalitis or occurring in a post-transplant setting, and neurological autoimmunity triggered by TNFα inhibitors or immune checkpoint inhibitors for cancer treatment. Awareness of the range of clinical and radiological manifestations associated with different neural autoantibodies, and the specific settings where autoimmune CNS disorders may occur is crucial to allow rapid diagnosis and early initiation of treatment.

## Introduction

The spectrum of immune-mediated neurologic disorders is rapidly expanding with the growing understanding of diverse disease mechanisms that can lead to central and/or peripheral nervous system inflammation (1, 2). Antibody-mediated disorders of the central nervous system (CNS) represent a distinct subgroup of immune-mediated neurologic disorders characterized by the presence of autoantibodies directed against specific neuronal or glial target antigens (generically referred as neural autoantibodies) mostly expressed in the CNS, and which share several distinctive clinical and MRI features. Novel neural autoantibodies continue to be discovered but “seronegative” autoimmune CNS disorders do occur in clinical practice as not all antibodies have been identified, and some autoimmune CNS disorders may be driven by predominant cell-mediated mechanisms not harboring an accompanying autoantibody (3). Clinical and MRI diagnostic criteria have been published to facilitate identification of these seronegative forms (1).

In this article, we will review the main clinical syndromes and MRI characteristics associated with neural autoantibodies directed against CNS antigens, and discuss commonly encountered challenges for diagnosis and management. Since not all target antigens are exclusively expressed in the CNS, certain neural autoantibodies are associated with complex clinical syndromes characterized by concomitant CNS and extra-CNS manifestations. On the contrary, neurological syndromes associated with autoantibodies exclusively targeting neuromuscular structures (e.g., myasthenia gravis) will not be discussed in this article; yet share many similarities in terms of pathophysiology and management.

### Principles of Autoimmune Neurology

Neural autoantibodies are generally classified on the basis of the cellular location of their target antigens, intracellular vs. cell-surface or synaptic (4). As a general rule, autoantibodies targeting intracellular antigens (e.g., nuclear, cytoplasmic) that are not accessible for antibody binding are thought not to be directly pathogenic but rather represent biomarkers of a predominantly cell-mediated cytotoxic process (5). On the contrary, antibodies targeting cell-surface or synaptic antigens act via direct binding to their target and are more likely to be directly pathogenic (6). Proposed mechanisms of antibody-mediated cell damage/dysfunction include blockade and internalization of the target antigen (7, 8), antibody-dependent complement activation and cellular cytotoxicity (9), inhibition of protein-protein interactions with subsequent loss of cellular connectivity (10), and disruption of the cytoskeletal architecture (11, 12). Autoantibodies targeting glial antigens (e.g., AQP4, GFAP, MOG) are predominantly associated with CNS demyelination or perivascular inflammation but may indirectly lead to neuronal loss via disruption of their oligodendrocytic or astrocytic targets (e.g., glutamate excitotoxicity secondary to AQP4 antibody-mediated astrocytic dysfunction) (13). For both neuronal and glial targets, clinically relevant neural autoantibodies are generally of the IgG1 and IgG3 subclasses (14), although with some such as CASPR2, LGI1, and IgLON5 the IgG4 subclass is frequent and may predominate (10, 15). Concomitant detection of more than one neural autoantibody may rarely occur (16).

The cellular location of the target antigens also has clinical implications. Antibodies targeting intracellular antigens are rare in children, have a stronger cancer association (onconeural antibodies), and generally respond less well to immunosuppressive treatments (17); while antibodies targeting cell-surface/synaptic antigens are better recognized to occur in both children and adults (18, 19), are less commonly paraneoplastic, and often respond well to immunosuppression (6), although exceptions exist for each group. Tables 1, 2 summarize the main antibodies targeting intracellular and cell-surface antigens, respectively, and their commonly associated neurological syndromes and cancers (if any).

**Table 1:** Demographic, clinical, and oncologic associations for neural autoantibodies targeting intracellular antigens.

**Neural antibody target**	**Typical age range and gender affected**	**Clinical syndrome**	**Cancer association % (cancer type)**
AK5	Age 60–70; possible male predominance	Limbic encephalitis	<10%
AGNA1(SOX1)	Age 60–70; no gender predominance	Lambert eaton myasthenic syndrome	>80% (SCLC)
Amphiphysin	Age 60–70; slight female predominance	Limbic encephalitis, stiff-person syndrome, cerebellar ataxia, myelopathy, polyradiculoneuropathy	>80% (SCLC, breast cancer)
ANNA-1/Hu	Age 60–70; slight female predominance	Limbic encephalitis, cerebellar ataxia, sensory neuronopathy and other neuropathies, dysautonomia, and gastrointestinal dysmotility, myelopathy (uncommon)	> 80% (SCLC, occasionally neuroblastoma in children)
ANNA-2/Ri	Age 60–70; slight female predominance	Brainstem syndrome (e.g., opsoclonus-myoclonus, jaw dystonia), cerebellar ataxia, peripheral neuropathy, myelopathy	>80% (SCLC, breast cancer)
ANNA-3	Age 50–60; 70% female	Limbic encephalitis, cerebellar ataxia, peripheral neuropathy, myelopathy	>80% (SCLC)
BRSK2	Unknown	Limbic encephalitis	>80% (SCLC)
CARP VIII	Age 60–80; possible female predominance	Cerebellar ataxia	>80% (Melanoma, ovarian tumors)
CRMP-5/CV2	Age 60–70; no gender predominance	Encephalitis, chorea, cerebellar ataxia, optic neuropathy, other cranial neuropathies, myelopathy, polyradiculoneuropathy	>80% (SCLC, thymoma)
GAD65	Age 50–60 (earlier in patients with epilepsy); 70% women	Limbic encephalitis, focal-onset seizures, stiff-person spectrum disorders, cerebellar ataxia	<10%
GFAP	Age 50–60; slight female predominance	Meningoencephalomyelitis, optic disc edema	25% (Ovarian teratoma)
GRAF/ARHGAP26	Unknown, adult age; possible female predominance	Cerebellar ataxia	Possible association with ovarian carcinoma
ITPR1	Age 60–70; 70% women	Cerebellar ataxia, peripheral neuropathy, encephalitis with seizures, myelopathy	30–40% (Breast cancer)
Kelch11	Age 40-50; 100% men	Ataxia, hearing loss, encephalopathy	>80% (Testicular seminoma)
LUZP4^*^	Age 40–50; >90% men	Rhombencephalitis, limbic encephalitis, seizures	>80% (Germ-cell tumors)
Ma2/Ta	Age 60–70 in women, 30–40 in men; male gender most affected (70%)	Some combination of limbic, diencephalic and brainstem encephalitis	>80% (Testicular germ-cell tumors in young males, lung cancer in other patients)
Neurochondrin	Age 40–50; possible female predominance	Cerebellar ataxia, brainstem dysfunction, myelopathy	<20%
NIF	Age 60–70; no gender predominance	Cerebellar ataxia, encephalopathy, myelopathy	75% [SCLC or other neuroendocrine (e.g., Merkel cell skin cancer)]
PCA-1/Yo	Age 60–70; strong female predominance and rarely seen in men	Cerebellar ataxia, peripheral neuropathy (uncommon), myelopathy (uncommon)	>80% (ovarian, fallopian tube, or breast carcinomas)
PCA-2/MAP1B	Age 60–70; 70% women	Encephalopathy, limbic encephalitis, cerebellar ataxia, polyradiculoneuropathy	>80% (SCLC)
PDE10A	Age 60–80; no clear gender predominance	Hyperkinetic movement disorders, parkinsonism	>80% (various)
Protein kinase C	Age 40–70; no clear gender predominance	Cerebellar ataxia	>80% (Non–small cell lung cancer, hepatobiliary adenocarcinoma)
TRIM46	Age 60–80; possible male predominance	Encephalitis, cerebellar ataxia	>60% (SCLC)
ZIC4	Age 60–70; male predominance	Cerebellar ataxia	>80% (SCLC)

* *Often coexist with Kelch11 autoantibodies*.

*AK5, adenylate kinase 5; AGNA1, Anti-glial nuclear antibody type 1; ANNA-1, Anti-neuronal nuclear antibody type 1; ANNA-2, Anti-neuronal nuclear antibody type 2; ANNA-3, Anti-neuronal nuclear antibody type 3; BRSK2, BB serine/threonine kinase 2; CARP VIII, Carbonic anhydrase-related protein VIII; CRMP5, collapsin response mediator protein-5; GAD65, glutamic acid decarboxylase-65; GFAP, glial fibrillary acidic protein; Kelch11, Kelch-like protein 11; LUZP4, Leucine zipper 4; MAP1B, microtubule-associated protein 1B; NIF, neuronal intermediate filament; PCA-1, Purkinje cell cytoplasmic antibody type 1; PCA-2, Purkinje cell cytoplasmic antibody type 2; PDE10A, phosphodiesterase 10A; SCLC, small cell lung cancer; TRIM46, tripartite motif 46*.

**Table 2:** Demographic, clinical, and oncologic associations for neural autoantibodies targeting extracellular cell-surface/synaptic antigens.

**Neural antibody target**	**Typical age range and gender affected**	**Clinical syndrome**	**Cancer association % (cancer type)**
AMPAR	Age 60–70; >70% women	Limbic/extra-limbic encephalitis	60% (SCLC, thymoma)
AQP4	Age 30–40; 90% women	Optic neuritis, myelitis, area postrema syndrome, encephalopathy (uncommon)	<20%
CASPR2	Age 60–70; >70% men	Limbic encephalitis, focal-onset seizures, episodic cerebellar ataxia, peripheral nervous system hyperexcitability +/– central and autonomic hyperxcitablility (Morvan's syndrome), neuropathic pain	<10% (Thymoma)
DPPX	Age 50–60; >60% men	Encephalopathy, brainstem syndromes, central nervous system hyperexcitability, gastrointestinal dysmotility, diarrhea, weight loss	<10% (B-cell malignancies)
GABA_A_R	Age 40–50; no gender predominance	Focal-onset seizures, encephalopathy, hyperkinetic movement disorders	25% (Thymoma)
GABA_B_R	Age 60–70; no gender predominance	Limbic encephalitis with refractory seizures	50% (SCLC)
GlyR	Age 40–60; no gender predominance	Progressive encephalomyelitis with rigidity and myoclonus, other stiff-person spectrum disorders	20% (Thymoma)
mGluR1	Age 50–60; no gender predominance	Ataxia, dysgeusia	20% (Lymphoma)
mGluR5	Age 20–30; no gender predominance	Limbic/extra-limbic encephalitis, focal-onset seizures, hyperkinetic movement disorders	60% (Hodgkin's lymphoma)
IgLON5	Age 60–70; no gender predominance	Sleep disturbances, bulbar symptoms, ataxia, chorea	<10%
LGI1	Age 60–70; >60% men	Limbic encephalitis, focal-onset seizures (e.g., faciobrachial dystonic seizures), sleep disturbance, hyponatremia	<10% (Thymoma)
MOG	Age any, children over-represented; no gender predominance	Acute disseminated encephalomyelitis, unilateral cortical encephalitis, optic neuritis, myelitis	<10%
Neurexin 3α	Age 40–50; possible female predominance	Encephalopathy, oro-facial dyskinesias, central hypoventilation	Unknown
NMDAR	Age 20–30; 80% women	Encephalopathy, speech disorders, seizures, dyskinesias, dysautonomia, hypoventilation	40% (Ovarian teratoma in females age 12–45; tumor is rare in males and females <12 years of age)
PCA-Tr/DNER	Age 60–70; >70% men	Cerebellar ataxia	>80% (Hodgkin's lymphoma)
VGCC (P/Q-type)^*^	Age 50–60; slight female predominance	Lambert eaton myasthenic syndrome	50–80% (SCLC)

* *Reported in association with ICI neurological irAE*.

*AchR, acetylcholine receptor; AMPAR, α-amino-3-hydroxy-5-methyl-4-isoxazolepropionic acid receptor; AQ4, aquaporin-4; CASPR2, contactin-associated protein-like 2; DNER, delta/notch-like epidermal growth factor–related receptor; DPPX, dipeptidyl-peptidase–like protein 6; GABA_A_R, gamma-aminobutyric acid (A) receptor; GABA_B_R, gamma-aminobutyric acid (B) receptor; IgLON5, immunoglobulin-like cell adhesion molecule 5; LGI1, leucine-rich glioma-inactivated 1; mGluR1, metabotropic glutamate receptor 1; mGluR5, metabotropic glutamate receptor 5; MOG, myelin oligodendrocyte glycoprotein; NMDAR, N-methyl-D-aspartate receptor; PCA-Tr, Purkinje cell cytoplasmic antibody Type Tr; SCLC, small cell lung cancer; VGCC, voltage-gated calcium channel*.

### Importance of Neural Autoantibody Detection in Clinical Practice

Identification of specific neural autoantibodies in patients with suspected autoimmune neurologic disorders provides important advantages to the clinician, including:

(1) Diagnostic confirmation—confirming a diagnosis of autoimmune neurologic disorder is crucial to avoid treatment delays, inappropriate treatments for other disorders, and unnecessary diagnostic procedures (e.g., brain biopsy) (1);

(2) Treatment precision—specific treatments often differ between different antibody-mediated disorders, but also from other immune-mediated disorders without specific neural autoantibodies. For instance, disease modifying agents commonly used to treat multiple sclerosis (e.g., interferon, fingolimod) may be ineffective or even worsen AQP4 and MOG antibody-associated disorders (20–22);

(3) Cancer prediction—certain neural autoantibodies are strongly associated with specific types of cancer, and can appear months to years before the cancer becomes clinically manifest allowing early diagnosis and treatment (16).

However, neural autoantibody testing is not flawless, and its reliability depends on a number of factors including the type of laboratory assay used for detection, the characteristics of the population tested, and the clinical setting.

## Diagnosis of Antibody-Mediated CNS Disorders

There are two major requirements for a correct diagnosis of antibody-mediated CNS disorder to be made: (1) a reliable identification of one or more specific neural autoantibodies; and (2) a compatible clinical-MRI phenotype.

### Identification of Compatible Clinical-MRI Phenotypes

**Figure 1:**
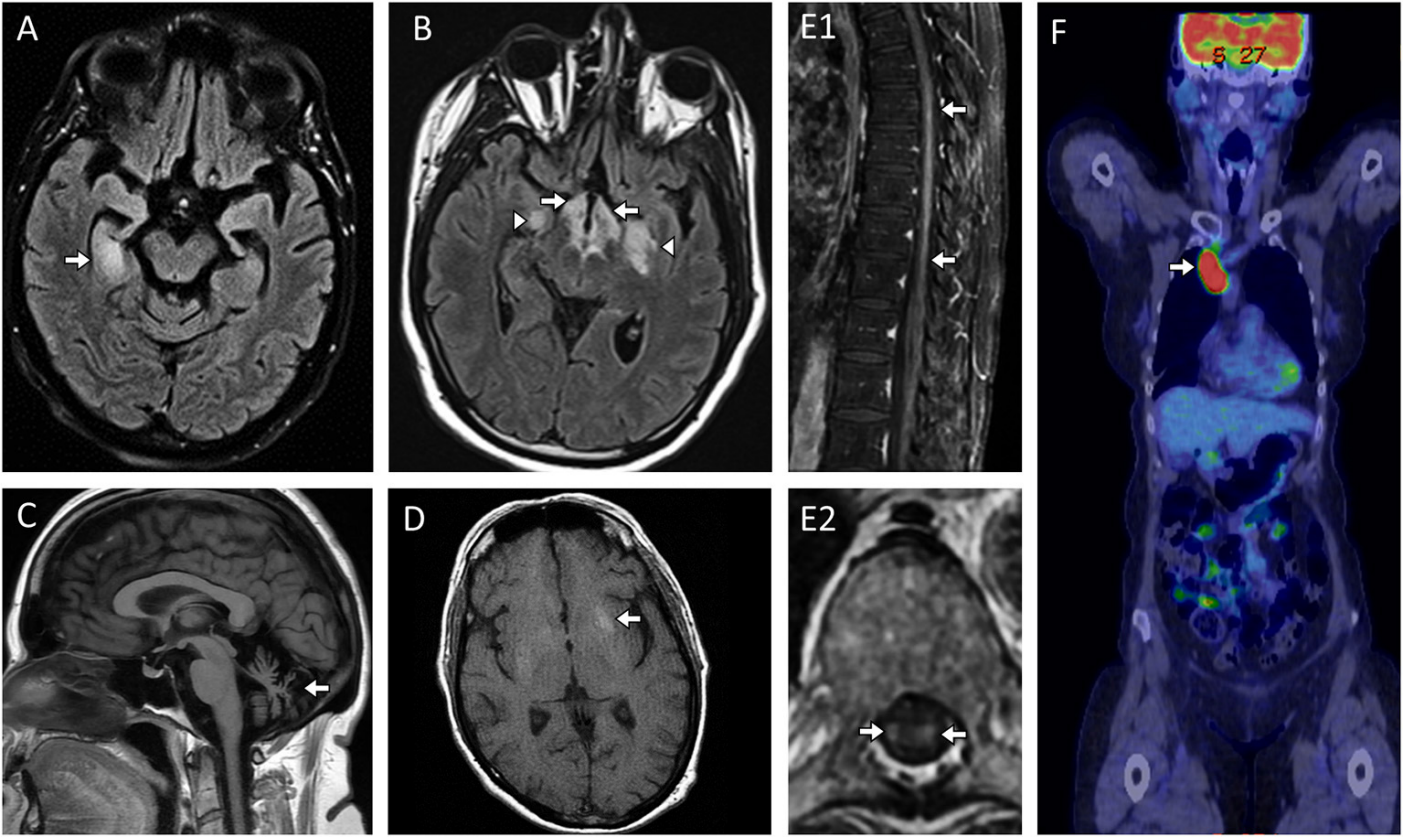
MRI abnormalities in patients with paraneoplastic and other non-demyelinating antibody-mediated CNS disorders. **(A)** Paraneoplastic limbic encephalitis with antineuronal nuclear antibody type 1 (ANNA-1/anti-Hu) with MRI head axial FLAIR image revealing unilateral T2-hyperintensity in the right mesial temporal lobe [**(A)**, arrow]; **(B)** Paraneoplastic narcolepsy-cataplexy and limbic encephalitis with Ma-2/Ta antibodies and MRI axial FLAIR image revealing bilateral T2-hyperintensity in the hypothalamus [**(B)**, arrows] and bilateral mesial temporal lobes [**(B)**, arrowheads]; **(C)** Progressive cerebellar ataxia with neurochondrin autoantibodies and MRI head sagittal T1-weighted images without gadolinium revealing severe cerebellar atrophy [**(C)**, arrow]; **(D)** Facio-brachial dystonic seizures with leucine-rich-glioma-inactivated-1 (LGI1) antibodies with MRI head axial T1-weighted images without contrast administration revealing T1-hyperintensity in the left basal ganglia [**(D)**, arrow]; **(E)** Paraneoplastic myelopathy accompanying small cell lung cancer without an identified neural autoantibody with MRI spine T1-weighted sagittal and axial images with gadolinium revealing enhancement within the spinal cord parenchyma [**(E1, E2)**: arrows] which on axial images was restricted to the bilateral lateral columns [**(E2)**, arrows) which is a hallmark imaging finding with paraneoplastic myelopathy; **(F)** Paraneoplastic encephalitis with α-amino-3-hydroxy-5-methyl-4-isoxazolepropionic acid (AMPA) receptor antibodies and 18F-Flludeoxyglucose-positron-emission-tomography (FDG-PET) body revealing markedly increased glucose uptake in the right paratracheal region [**(F)**, arrow] with subsequent biopsy revealing metastatic small cell lung cancer.

Except for some highly suggestive syndromes, the clinical manifestations of antibody-mediated CNS disorders are often nonspecific (e.g., encephalopathy, status epilepticus) and indistinguishable from those of other non-inflammatory etiologies (e.g., metabolic, neoplastic, infectious) so that exclusion of alternative and more common diagnoses is mandatory (23–25). An acute to subacute onset of neurological deficits over days to few weeks is characteristic and should always raise the suspicion for an autoimmune CNS disorder (1), although a more slowly progressive clinical presentation can be seen with certain autoantibodies (e.g., DPPX, GFAP, and IgLON5 autoantibodies) (26–28). At nadir, the clinical syndromes are frequently severe and often involve multiple functional neurologic domains (29). For instance, focal epileptic seizures or behavioral changes may occur in isolation at onset but often evolve rapidly to status epilepticus and/or encephalopathy, which can help diagnostically to distinguish from isolated epilepsy, psychiatric disorders, or typical neurodegenerative dementia (30–32). Different scoring systems have been designated to help clinicians determine the likelihood of autoantibody detection in patients with different neurological manifestations (33–35). Viral-like prodromes or vaccinations are not uncommon at or before symptom onset (26–28, 36). An inflammatory CSF with >5 white blood cells/mm^3^ with or without oligoclonal bands is useful to help distinguish from non-inflammatory mimics when present, but its frequency might decrease in the elderly or depend on the antibody associated (37). The detection of specific abnormalities on brain and/or spinal cord MRI may support the diagnosis and help differentiation from other etiologies, although a normal MRI is well-recognized with a number of neural antibodies (23). Examples of MRI abnormalities seen with different neural autoantibodies are shown in Figure 1 and have been reviewed in detail recently elsewhere (38).

#### Paraneoplastic and Non-demyelinating Syndromes Suggestive for an Antibody-Mediated Etiology

*Limbic encephalitis* is one of the most common phenotypes and can be seen in association with a wide variety of neural autoantibodies (Tables 1, 2) (39). It is characterized by acute-subacute onset of short memory impairment, behavioral changes, altered mental status, and/or seizures (40). Brain MRI often shows unilateral or bilateral T2-hyperintensity of the mesial temporal lobes (Figures 1A,B), with or without associated enhancement. The main differential diagnosis is with herpes simplex virus encephalitis (41), neoplasms (42), and seizures (43). Autoimmune limbic encephalitis is more commonly bilateral and infrequently extends outside the mesial temporal lobes, although acute distinction can be challenging based on MRI alone.

*Progressive encephalopathy with rigidity and myoclonus (PERM)* is a rare phenotype characterized by the occurrence of muscular rigidity, spasms, and myoclonus in association with encephalopathy and usually accompanied by a normal MRI head and spine (44). This syndrome is part of the stiff-person spectrum disorders and is mostly seen with GAD65 and GlyR autoantibodies; autoimmune cerebellar ataxia and epilepsy may also coexist with GAD65 antibodies (45, 46). Tetanus and malignant hyperthermia are among the differential diagnosis.

*Morvan syndrome* is defined by the co-occurrence of encephalopathy and peripheral nerve hyperexcitability (irregular muscle contractions, fasciculations, and cramping), insomnia, hyperhidrosis and is frequently seen with LGI1 and Caspr2 autoantibodies (47, 48).

*Facio-brachial dystonic seizures (FBDS)* are short-lasting dystonic movements primarily affecting the arm and face on one side, with a high daily frequency (up to 40–60 times per day) (49). These seizures are highly specific for LGI1 autoantibodies and can be accompanied by basal ganglia hyperintense lesions on T1 (Figure 1D) or T2-weighted sequences without gadolinium (50).

*Opsoclonus-myoclonus syndrome* is characterized by rapid, multi-directional eye movement and diffuse muscle jerks, often in association with ataxia. This syndrome can be seen in children with neuroblastoma and ANNA1/anti-Hu antibodies but also in adults with breast or lung cancers and ANNA2/anti-Ri antibodies (51, 52). Laryngospasm and neck or jaw-opening dystonia can also occur with the paraneoplastic neurologic syndrome accompanying ANNA-2/anti-Ri antibodies (52).

*Multifocal, large cortico-subcortical lesions* on brain MRI in association with refractory status epilepticus are typically found with antibodies targeting GABA_A_R and may occur in children (53). A similar MRI appearance is seen with MELAS (mitochondrial encephalomyopathy, lactic acidosis, and stroke-like episodes) (54).

#### Demyelinating Syndromes Suggestive for an Antibody-Mediated Etiology (Figure 2)

**Figure 2:**
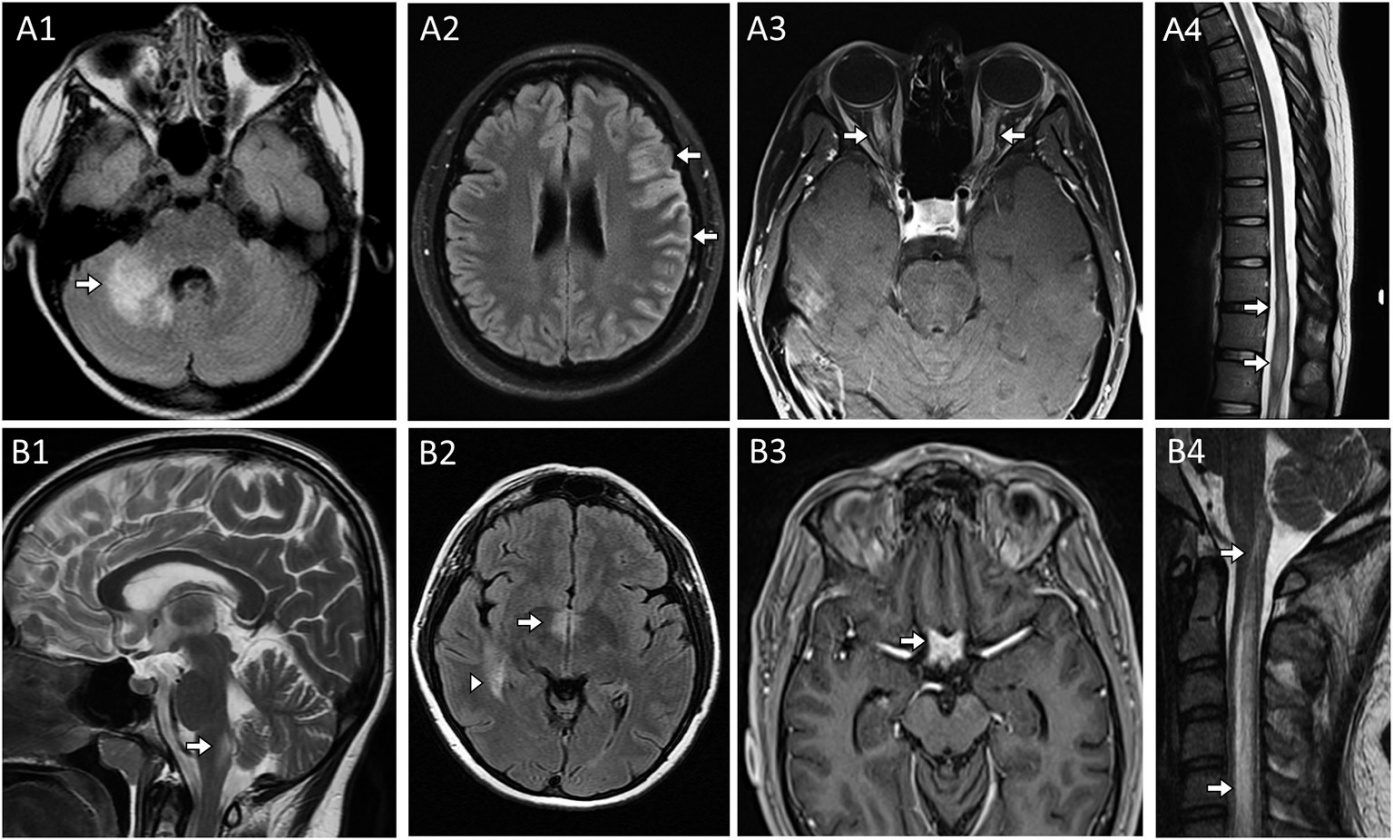
MRI head, orbit, and spine examples in patients with CNS demyelinating disease from **(A)** Myelin Oligodendrocyte Glycoprotein-IgG antibody associated disorder and **(B)** Aquaporin-4-IgG seropositive neuromyelitis optica spectrum disorder. **(A)** Myelin Oligodendrocyte Glycoprotein-IgG (MOG) autoantibody associated disorder (MOGAD). Head MRI axial FLAIR images revealing characteristic large unilateral T2-hyperintense lesion in the right middle cerebellar peduncle with indistinct margins [**(A1)**, arrow] in a patient a cerebellar attack and unilateral cortical T2-hyperintensities and swelling [**(A2)**, arrows] in a patient with a unilateral cortical encephalitis attack. MRI orbits axial post-gadolinium images revealed bilateral anterior optic nerve enhancement with swelling [**(A3)**, arrows] in a patient with bilateral optic neuritis. Thoracic spine MRI sagittal T2-weighted image reveals characteristic T2-hyperintensity with swelling within the conus [**(A4)**, arrow] in the setting of a transverse myelitis attack. **(B)** Aquaporin-4-IgG seropositive neuromyelitis optica spectrum disorder. Sagittal T2-weighted brain image reveals T2-hyperintensity in the dorsal medulla adjacent to the 4th ventricle [**(B1)**, arrow] in in a patient with an area postrema attack resulting in intractable nausea, vomiting and hiccups; MRI head axial FLAIR image reveals T2-hyperintensity adjacent to the 3rd ventricle [**(B2)**, arrow] and an additional T2-hyperintense lesion in the medial temporal lobe [**(B2)**, arrowhead]; MRI of the head and orbits with axial T1-weighted image post-gadolinium reveals enhancement of the optic chiasm [**(B3)**, arrow]; Cervical spine MRI sagittal T2-weighted image reveals a longitudinal extensive T2-hyperintense lesion extending more than 3 vertebral segments [**(B4)**, arrows] in a patient with a transverse myelitis attack.

*Acute disseminated encephalomyelitis (ADEM)* or ADEM-like phenotypes are characterized clinically by encephalitis and/or myelitis accompanied by large T2-hyperintense demyelinating lesions in multiple CNS regions on MRI (spinal cord, brain, and/or optic nerves). Large T2 hyperintensities with indistinct margins can be encountered and involve the middle cerebellar peduncle (Figure 2A1) in the infratentorial region or deep gray matter in the supratentorial region (19, 55–57). MOG antibodies are found in 30–50% of ADEM patients while AQP4 autoantibodies account for a much smaller proportion of cases (<5%) (2, 19, 58). In contrast to multiple sclerosis, oligoclonal bands are detected in only a minority (<20%) of patients with these autoantibodies (59–61).

*Area postrema syndrome*, characterized by intractable nausea, vomiting, and/or hiccups, from involvement of the vomiting center where aquaporin-4 is particularly enriched and accompanying T2-abnormalities in the dorsal medulla (Figure 2B1) are typical of this syndrome which associated with AQP4 autoantibodies (62). AQP4 is also enriched around the 3rd ventricle where T2-lesions also occur (Figure 2B2).

*Optic neuritis* is non-specific and may occur in association with different neural autoantibodies, but is particularly common in patients with antibody-mediated CNS demyelination, especially when recurrent and/or bilateral. An anterior optic neuritis involving >50% of the optic nerve length is more common with MOG antibodies (Figure 2A3); while involvement of the optic chiasm and posterior segment of the optic nerves is typical of AQP4 antibodies (Figure 2B3) (63, 64).

*Myelitis lesions* that are longitudinally-extensive (spanning ≥3 contiguous vertebral body segments on T2-weighted images on spine MRI) are also typical of both AQP4 antibodies (Figure 2B4) and MOG autoantibodies and may occur in isolation, accompanied by unilateral or bilateral optic neuritis (neuromyelitis optica), or concomitant brain involvement (65, 66). Conus involvement (Figure 2A4) can be a clue to MOG antibody-associated disorder and neurogenic bowel, bladder, and/or sexual dysfunction are common in conjunction with this (67, 68). Spinal cord MRI can be initially normal in myelitis patients with MOG autoantibodies (69). GFAP autoantibodies are also associated with longitudinally extensive spinal cord involvement but generally in the context of a meningo-encephalo-myelitis (70). Among non-demyelinating antibody-mediated CNS disorders, longitudinally extensive tract-restricted MRI abnormalities on T2 and T1-post gadolinium sequences, particularly along lateral columns (Figure 1E), are characteristic of the rare paraneoplastic myelopathies and most often accompanied by CRMP5/anti-CV2 or amphiphysin autoantibodies (71).

*Unilateral cortical FLAIR-hyperintense lesions in anti-MOG-associated encephalitis with seizures (FLAMES)* is a less common, but quite characteristic clinical-MRI phenotype seen with MOG autoantibodies (72, 73). Patients typically present with headache, fever, unilateral cortical deficit (e.g., aphasia, hemisensory loss) and seizures, accompanied by cortical enlargement and hyperintensity on FLAIR MRI sequences (Figure 2A2), sometimes with concomitant leptomeningeal enhancement (74). CSF pleocytosis is found in the vast majority of these patients, and can by very high mimicking an infectious process.

Other less specific syndromes include a diencephalic syndrome of narcolepsy and cataplexy with Ma2 antibody encephalitis (Figure 1B) or a brainstem encephalitis which can associate with Ma2 or Kelch-like-11 autoantibodies with hearing loss, vertigo and tinnitus a clue to the latter; both are associated with testicular tumors (75, 76). A progressive cerebellar ataxia syndrome can occur with a wide range of neural autoantibodies (Tables 1, 2) which may over time lead to cerebellar atrophy (Figure 1C). When this occurs in the setting of an underlying cancer, the term paraneoplastic cerebellar degeneration is used (77); rarely an episodic ataxia has been reported in association with CASPR2 antibodies (78), although needs to be distinguished from paroxysmal dysarthria-ataxia associated in multiple sclerosis (79). Chorea and hyperkinetic movement disorders with basal ganglia T2-abnormalities on MRI may occur with PDE10A and CRMP5 autoantibodies (80, 81); while non-REM sleep disturbances are characteristically seen with autoantibodies targeting IgLON5, sometimes in association with progressive bulbar dysfunction and muscular fasciculations that can mimic progressive supranuclear palsy or amyotrophic lateral sclerosis (27, 82). Lastly, new onset refractory status epilepticus (NORSE) is highly suggestive of CNS autoimmunity and neural autoantibodies directed against either cell-surface or intracellular targets can be detected in approximately one third of patients (83).

### Supportive Paraclinical Findings

Electroencephalography in patients with encephalitis is generally non-specific showing diffuse or focal slowing or epileptiform activity. A more characteristic pattern of rhythmic delta activity at 1–3 Hz with superimposed beta bursts at 20–30 Hz (extreme delta brush) is found in up to one third of patients with NMDAR autoantibodies (84), although it can also be seen in patients with non-autoimmune temporal lobe epilepsy, hypoxic-ischemic encephalopathy, and brain neoplasms (85). The facio-brachial dystonic seizures seen with LGI1 autoantibodies typically show no EEG correlate during episodes possibly from their short-lasting nature or potentially a deep origin in the basal ganglia (86).

18F-fluorodeoxyglucose positron emission tomography (FDG-PET) is more sensitive than brain MRI in patients with autoimmune encephalitis and can show abnormal hypometabolism, hypermetabolism, or both in a diffuse fashion or restricted to certain brain areas (e.g., mesial temporal poles) (87). Brain or spinal cord biopsy can be considered in uncertain cases without neural autoantibodies detectable to confirm an inflammatory origin of the neurological deficit.

### Cancer Search

In general, a CT chest, abdomen and pelvis is used as the initial screen for cancer. FDG-PET can help identify occult cancers before they become clinically manifest in patients with onconeural autoantibodies (Figure 1F) and may improve the sensitivity beyond CT in those in whom there is a high suspicion for a paraneoplastic neurologic disorder (88, 89). Sex-specific tests (*e.g*., mammogram, gynecologic/scrotal ultrasound) should not be overlooked. Certain autoantibodies have a strong association with specific types of cancer (Tables 1, 2) and their detection should prompt cancer screening (17). Based on this, the investigations to consider when assessing for cancer are summarized in Table 3.

**Table 3:** Cancer investigations to consider in patients with neural autoantibodies with a high likelihood of an accompanying cancer.

**Tumor**	**Investigations to consider**
Breast carcinoma	Mammogram, ultrasound, MRI, CT body, PET-CT body for metastases
Gynnecologic cancers (ovary, fallopian tube, uterine)	Ultrasound or CT of pelvis, PET-CT body, serum cancer antigen 125
Lymphoma	CT body, PET-CT body
Lung cancer (typically small-cell)	CT chest, PET-CT body
Melanoma, merkel cell carcinoma	Skin examination, CT body, PET-CT body
Neuroblastoma	Urine/serum catecholamines vanillylmandelic acid (VMA), homovanillic acid (HVA), metaiodobenzylguanidine (MIBG) scan, CT or MRI body
Teratoma (ovarian or other)	Transvaginal pelvic ultrasound, CT pelvis, MRI pelvis, CT chest, and abdomen for teratoma beyond pelvis
Testicular tumors (seminoma or other)	Scrotal ultrasound, CT body, PET-CT body, serum β-human chorionic gonadotropin, α-fetoprotein, lactate dehydrogenase
Thymoma/thymic carcinoma	CT chest

*CT, computed tomography; MRI, magnetic resonance imaging; PET-CT, 18F-Fludeoxyglucose-positron-emission-tomography-computed-tomography*.

### Neural Autoantibody Testing

**Figure 3:**
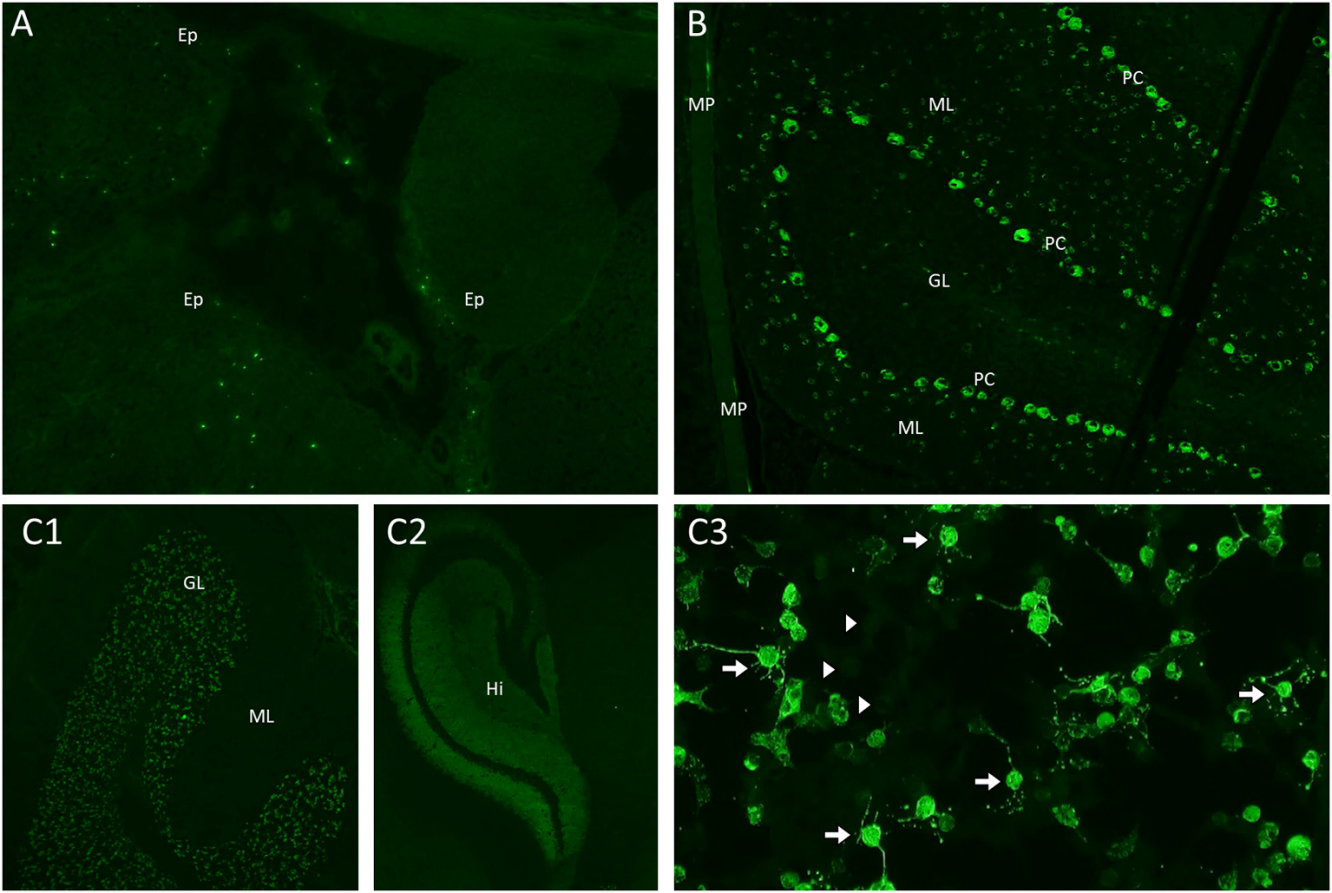
Mouse brain, gut, and kidney tissue immunofluorescence and cell-based assay assessment for neural autoantibodies. **(A)** Kelch-like-protein-11 autoantibodies are identified using a mouse tissue composite with immunofluorescence to identify the characteristic peri-ependymal (Ep) sparkles of immunostaining; **(B)** Purkinje-cell-autoantibody-type 1 (PCA-1/anti-Yo) antibodies are identified using a mouse tissue composite with immunofluorescence staining in the characteristic pattern with staining purkinje cells (PC), additional cells in the molecular layer (ML) of the cerebellum and the myenteric plexus (MP); **(C)** N-methyl-D-aspartate (NMDA) receptor antibodies are identified using a mouse tissue composite with the characteristic pattern of immunostaining involving the granular layer (GL) **(C1)** and hippocampus (Hi) **(C2)**; A confirmatory cell-based assay reveals characteristic immunostaining of cells transected with the NMDA receptor [**(C3)**, arrows] in comparison to non-transfected cells [**(C3)**, arrowheads].

Reliability of autoantibody positive results depends on the assay and biospecimen (serum vs. CSF) used for testing, and the setting in which the test is performed (90). Different laboratory assays bear different specificity and sensitivity based on the autoantibody tested and its characteristics, and not all the assays are appropriate for all existing neural autoantibodies (91). While a detailed description of the laboratory assays commonly used for autoantibody testing is beyond the scope of this article, it has recently been summarized elsewhere (90). Most autoantibodies are initially identified by a technique named tissue-based immunofluorescence (TIF). For this assay, a composite of different sections of animal (e.g., mouse) brain and non-brain tissue (which share a proportion of antigens with the human tissue) are incubated with the serum and/or CSF of the patient tested. If neural-tissue specific autoantibodies are present (i.e., binding the brain sections but not the non-brain ones), they can be detected by using a secondary anti-human immunoglobulin antibody marked with a fluorescent agent and visible on fluorescence microscopes. Based on the distribution of the target antigens on the different brain sections (e.g., selective staining of the hippocampus, cerebellum, or combinations), different staining patterns can be recognized (Figure 3). As a consequence, a big advantage of TIF is the possibility of identifying as yet unrecognized neural-tissue restricted staining patterns, which is the base for autoantibody discovery. Once a known staining pattern is recognized on the fluorescence microscope by the reader, a secondary assay is generally used for confirmation. The most utilized confirmatory assays are:

#### Cell-Based Immunofluorescence (Live or Fixed)

Human embryonic kidney (HEK) cells are transfected with genes encoding the target antigen of interest, which is expressed on the surface of the cell membrane. This technique allows binding of the human autoantibodies to their target antigen in its native conformational form and is generally the assay of choice for antibodies targeting cell surface/synaptic antigens (Figure 3C3) (92, 93). This technique may be used both to confirm an assay detected on TIF or be used in isolation, particularly for antibodies not reliably detected on TIF such as AQP4-IgG in which the specificity and positive predictive value are very high with this technique (>99%) despite being confirmed on just a single assay (94). Live cell-based assays have been shown to bear higher sensitivity and specificity than assays using fixed cells for detection of different autoantibodies, including AQP4-IgG and MOG-IgG (94, 95). This is likely due to reduced affinity of the autoantibodies for their target antigen after its tridimensional conformation has been altered by the fixation process.

#### ELISA, Western or Line Blot, and Radio-Immuno-Precipitation

These techniques allow recognition of solubilized antigens by their specific human antibodies but not in a native conformational form and are commonly used to detect antibodies targeting intracellular antigens (96, 97). These older-generation techniques have an increased risk of false positivity when used in isolation and care is advised with interpretation of isolated western or line blot results in particular (98, 99).

#### Cell Cultures (Less Common)

Live neuronal cell cultures can also be used to confirm autoantibody binding to surface antigens (100).

Unfortunately, TIF is routinely available in only few specialized laboratories worldwide and thus many laboratories utilize single methods for detection. Novel cell-based assays are now available which have much improved sensitivity and specificity and have a low risk of false positive results (0.2%) when compared to older generation techniques in which false positives are much more common (up to 6%) (91).

When possible, neural autoantibodies should be tested in both serum and CSF to maximize sensitivity, although certain autoantibodies are preferentially detected in either one of these biospecimens (101). NMDAR autoantibodies for instance are most sensitive and specific when detected in the CSF (102), while AQP4, MOG, and LGI1 autoantibodies have optimal sensitivity and specificity when tested in serum (103). Isolated CSF positivity has been reported in patients with CNS demyelination associated with MOG autoantibodies and typical clinical-MRI phenotype (104). The timing of testing is also important for diagnostic accuracy. When possible, samples should be obtained before immunosuppressive treatment and during attacks, as antibody titer may drop to undetectable after immunosuppression and/or during disease remission. Passive transfer of low titer autoantibodies commonly found in the general population and of low clinical significance (e.g., low titer GAD65 autoantibodies) may occur with infusion of hemoderivatives (e.g., immunoglobulins) and should be kept in mind when antibody testing is performed after administration of such products (105).

### Positive Predictive Value, False Positives, and “Phenotype Creep”

**Figure 4:**
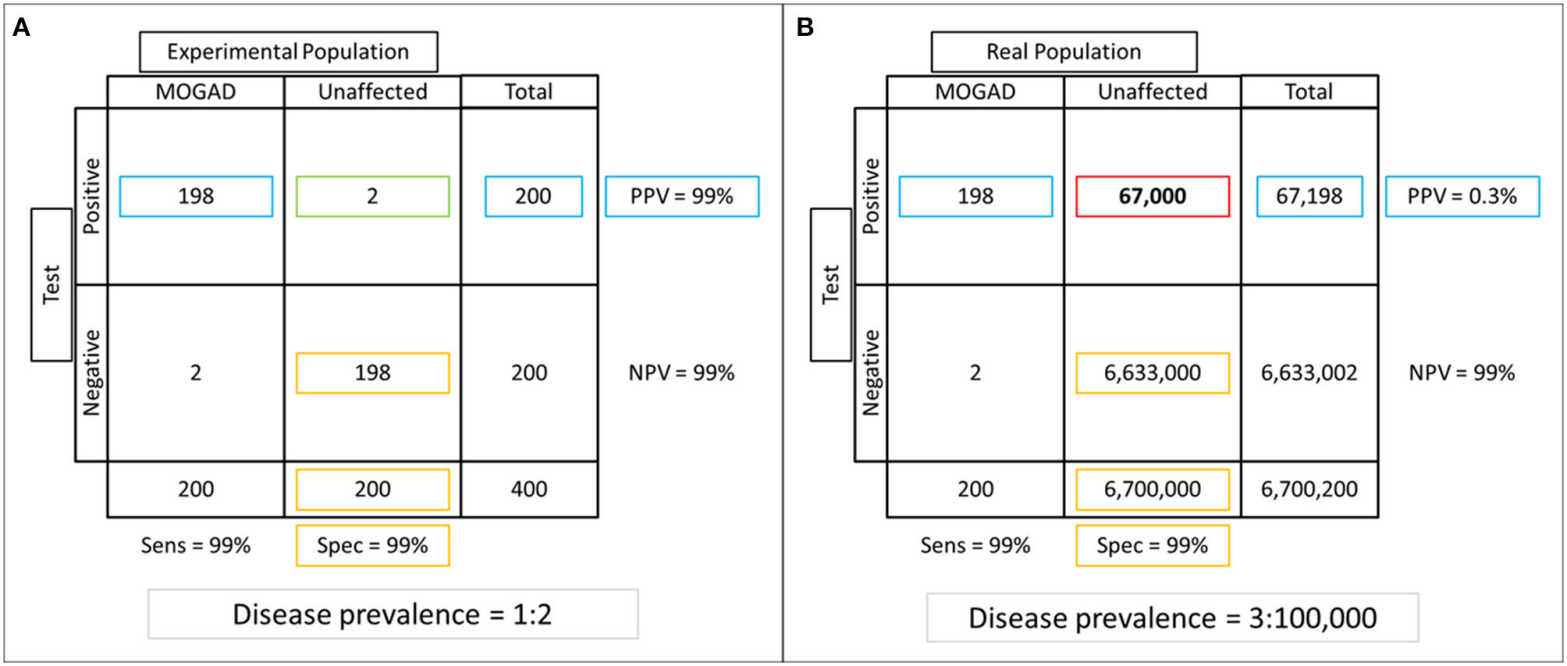
Example of specificity and positive predictive value (PPV) assessment in experimental vs real-life settings. **(A)** An experimental population composed of equal number of patients with the disease of interest and unaffected controls is tested (frequency of the disease of interest in the tested population = 50%). Of 200 positive results obtained after autoantibody testing, 2 (1%) are false positives (green box), while the majority of those who tested negative were true negatives (*n* = 198), for a specificity of 99% (true negatives divided by total unaffected patients; yellow boxes). The PPV (number of true positives divided by total positive results) is also very high in this setting (99%; blue boxes). **(B)** If the same test is performed in a hypothetical metropolis of 6,700,200 inhabitants where the disease prevalence is 0.003%, notice that the number of false positive results increases dramatically to 67,000 (red box). While this variation in frequency of false positive results does not affect specificity (99%, yellow boxes), which is an intrinsic characteristic of the test and thus not affected by the characteristics of the population tested, it has a huge impact on the PPV that decreases to 0.03% (blue boxes).

Despite a high accuracy reported for most of the commercially available assays, a positive autoantibody result does not necessarily imply that the patient has an autoimmune neurologic disorder. As with any diagnostic biomarker, sensitivity, and specificity are intrinsic characteristics of the test and measure the quality of the assay and the testing laboratory, but are less reflective of the setting in which the test is performed. A more useful parameter in clinical practice is the positive predictive value (PPV), which is the ratio between true positives and total positive results obtained by testing a given population, and is highly dependent on the frequency of the disease of interest in the population tested and the test ordering practices (106). This is particularly relevant for rare disorders like autoimmune neurologic disorders, for which the reported overall incidence and prevalence range between 3.5 and 9/million person-years and 4–6.5/100.000 persons, respectively (107–109). When similar rare disorders are faced, caution is advised in clinical practice as the risk of false positive results is not negligible if the test is performed indiscriminately, despite very high specificity and sensitivity (110). The example in Figure 4 illustrates how the PPV can vary when a given autoantibody is tested in different populations. When the autoantibody of interest is tested in a controlled experimental setting (Figure 4A) where the same amount of affected and unaffected patients are tested, both the specificity and PPV of the test are 99% with a very low rate of false positive results (1%). On the contrary, when the same test is performed indiscriminately in a hypothetical metropolis of nearly 7 million inhabitants and a prevalence of the disease of interest of 3/100,000 (Figure 4B), the PPV drops dramatically to 0.3% with a disproportionally high number of false positive results (67,000). Despite its surreal nature (screening of an entire metropolis would be highly unlikely to occur in real-life), this serves as an extreme example of the potential consequences of indiscriminate testing for a rare disorder in a high throughput setting. The same concept is applicable in clinical practice where antibody-mediated CNS disorders are far less common than other immune-mediated and non-immune-mediated neurologic diseases (e.g., multiple sclerosis in the extremes of latitude, epilepsy, cancers). Thus, a thorough assessment of the clinical-MRI phenotypes is mandatory when autoantibody testing is performed, as “atypical” or “rare” manifestations for a given antibody-associated syndrome should prompt investigating alternative etiologies (which are simply more likely to occur than rare manifestations of a rare disorder) (110). The concept of “phenotype creep” has been coined to specifically describe the incorrect tendency in the scientific literature and clinical practice to accept poorly fitting manifestations as an uncommon part of the disease spectrum, just based on autoantibody positivity (111).

High autoantibody titers are less likely to be seen with false positive results, but they still do not guarantee 100% specificity. GAD65 autoantibodies are a typical example since they can be non-specifically found at low titers in diabetic patients or patients without neurologic deficits. However, the detection of high-titer GAD65 autoantibodies in the serum and/or CSF of neurologic patients is not sufficient for a diagnosis of GAD65 autoimmunity in the absence of one of the compatible clinical phenotypes of stiff-person spectrum, autoimmune ataxia or autoimmune epilepsy. In a recent study of 323 patients with high-titer GAD65 autoantibodies seen at Mayo Clinic, one third had an alternative diagnosis (45). Awareness of the typical clinical-MRI phenotypes associated with the different neural autoantibodies is important to avoid misdiagnosis and inappropriate treatment. In case of uncertainty, external consultation from autoimmune neurology experts or the reporting neuroimmunology laboratory is advised.

### Available Diagnostic Criteria

Specific diagnostic criteria have been published to guide clinicians during the diagnostic process in patients with suspected autoimmune encephalitis (1). Based on these criteria, the concomitant detection of a number of typical clinical and paraclinical features for autoimmune encephalitis (e.g., subacute onset, CSF pleocytosis, bilateral mesial temporal lobe T2-hyperintensity on brain MRI) allows a possible or definite diagnosis independently of neural autoantibody detection (1). Such clinical-MRI phenotypical requirements reduce the risk of false positive results and are often used as a clinical gold standard in research to assess antibody specificity and sensitivity (1).

Among autoimmune demyelinating CNS disorders, international consensus diagnostic criteria exist for neuromyelitis optica spectrum disorders (NMOSD) with AQP4 autoantibodies (66). Although preliminary diagnostic criteria and recommendations have been proposed for MOG antibody associated disorder (19, 112), there are international efforts to produce more unified consensus diagnostic criteria. These criteria will be important, given there is a higher risk of false positives, particularly at low titer with MOG antibody associated disorder than with aquaporin-4 antibody positive NMOSD (110).

### Neurological Syndromes Associated With Systemic Autoantibodies

Otherwise unexplained neurological manifestations in patients with systemic autoimmune/inflammatory disorders have frequently been regarded as being part of the systemic syndrome, especially in the past. Although neurological manifestations of systemic disorders are possible (e.g., stroke from intracranial vessel involvement in patients with systemic vasculitis) (113), the concomitant occurrence of multiple autoimmune disorders in the same patient is probably more likely (114). For this reason, neural autoantibody testing is always recommended in patients with neurological manifestations and systemic inflammatory disorders (e.g., systemic lupus erythematosus, Sjogren disease) (115). Hashimoto encephalopathy is a historical disease entity characterized by steroid-responsive subacute encephalopathy and high titer anti-thyroid autoantibodies in serum. Despite the lack of a convincing pathogenetic mechanism linking anti-thyroid antibodies and encephalopathy, Hashimoto encephalopathy is still considered a diagnostic possibility by many clinicians. Recent studies from different international groups have shown this association is spurious and anti-thyroid antibodies can be detected with similar frequency in both patients with antibody-mediated encephalitis, seronegative forms, and non-autoimmune etiologies (1, 116, 117).

### Emerging Clinical Settings Associated With Autoimmune CNS Disorders

Specific clinical settings are increasingly recognized to be at higher risk for development of autoimmune CNS disorders. These include:

#### Post-herpes Simplex Virus Encephalitis

HSV encephalitis often present with symptoms/signs of mesial temporal lobe involvement and can be difficult to differentiate from autoimmune limbic encephalitis. However, autoimmune encephalitis may develop after HSV encephalitis (generally within 3 months) in up to 27% of patients. These forms are generally associated with neural autoantibodies (frequently targeting NMDAR) and should be suspected in patients with HSV encephalitis who show clinical and/or MRI worsening after an initial improvement with anti-viral therapy (118).

#### Post-transplant Autoimmunity

Autoimmune CNS disorders may rarely paradoxically arise in post-transplant patients despite ongoing immunosuppressant medication use to prevent organ rejection. It is possibly due to imbalance between B- and T-cell as anti-rejection immunosuppression are predominantly T-cell targeting treatments. Testing neural antibodies in this scenario is reasonable and the addition of antibody depleting treatments (plasma exchange, rituximab) in addition to maintenance anti-rejection immunosuppression should be considered (119).

#### CNS Inflammation Secondary to TNFα Inhibitors

TNFα inhibitors are used for treatment of many systemic (e.g., Crohn's disease) and neurologic (e.g., neurosarcoidosis, Behcet disease) chronic inflammatory disorders. CNS inflammatory disorders of any type, with or without accompanying neural autoantibodies, may develop in patients treated with these drugs (generally during the first year) with a higher frequency compared to untreated patients (120).

#### Neurological Autoimmunity Secondary to Immune Checkpoint Inhibitors

ICI act by enhancing the immune response against cancers and are increasingly approved for treatment of different tumors. A common and unwanted effect of these drugs is autoimmunity, potentially directed against any organ (121–125). CNS autoimmunity may occur, generally within the first 3 months, and is accompanied by neural specific autoantibodies in approximately half of moderate-high severe cases. Unlike their idiopathic counterparts, ICI-related antibody-mediated disorders seem characterized by a higher frequency of unclassified neural-tissue specific autoantibodies, and loss of the typical cancer-autoantibody paraneoplastic associations (e.g., autoantibodies typically detected with gynecological cancers can be found in patients with skin melanoma) (126).

#### Other Immune-Active Agents

Cases of neurological autoimmunity including antibody-associated encephalitis has been reported in multiple sclerosis patients treated with the CD52 (e.g., alemtuzumab) or CD25 (e.g., daclizumab) protein inhibitors (127–129).

#### Neurologic Autoimmunity Related to SARS-CoV-2

Diverse neurologic manifestations have been described in patients with SARS-CoV-2 infection during the COVID-19 pandemic, including antibody-associated CNS disorders (130, 131). Although this association remains controversial, viral or bacterial infections (commonly 10–20 days before symptoms onset) are often recognized as potential triggers in patients with neurologic autoimmune disorders via different mechanisms (e.g., molecular mimicry). On the contrary, a causal relationship between onset of neurological autoimmunity and vaccinations is more debated.

## Treatment and Outcomes

Current treatment strategies for antibody-mediated neurologic disorders are mostly based on anecdotal experience, expert opinion and data from retrospective case series. The treatment approaches differ based on the specific autoantibody detected, associated syndromes, and phase of the disease (acute/active CNS inflammation vs. remission). The rarity of these disorders has hampered the development of randomized controlled clinical trials in autoimmune neurologic disorders. However, the recent successful randomized controlled clinical trials of a number of attack-prevention targeted treatments in AQP4-IgG seropositive NMOSD has provided a platform for future clinical trials in antibody mediated syndromes. However, for most autoimmune neurologic disorders there are no proven treatments but some general principles have been developed that can guide treatment decisions when definitive scientific evidence is lacking.

### Acute Immunotherapy

The treatment approach for acute exacerbations of antibody-mediated CNS disorders is similar for the different underlying autoantibodies and includes one or more of:

*Intravenous methylprednisolone* [IVMP−1g daily for 3–5 days, based on the severity of symptoms and comorbidities (e.g., diabetes)]. The equivalent dose of 1,250 mg of prednisone once daily for 5 days can be considered as an alternative and has been utilized in some patients when there have been difficulties in arranging intravenous infusions (e.g., concern for attending medical facilities during the COVID 19 pandemic). Due to the lower risk of potential adverse effects, IVMP is generally preferred and often sufficient to improve symptoms as a monotherapy. Common adverse events related to IVMP include hyperglycemia in patients with diabetes or impaired glucose tolerance, and corticosteroid-induced psychosis.

*Intravenous immunoglobulins (IVIg–0.4 g/Kg once daily for 5 days)*.

*Plasma exchange* (PLEX–1 exchange every other day for 5–7 exchanges).

IVIg and PLEX are generally used as add-on to IVMP for more severe cases. IVIg may be contraindicated in patients with kidney disease, IgA deficiency, and is associated with an increased risk of thrombosis while PLEX may induce hypotension and should be avoided in patients with low baseline blood pressure and is complicated by the frequent need for central line placement. PLEX has been shown to be highly effective as acute monotherapy in patients with severely disabling idiopathic inflammatory diseases of the CNS with a randomized, sham-controlled, double-blinded trial. The study was published in 1999 and is likely that many of included patients with severe attacks of demyelination considered idiopathic at the time now would test positive for AQP4 or MOG autoantibodies (132). More recent data support the utility of PLEX in addition to IVMP for attacks of demyelination associated with AQP4 autoantibodies, especially within the first 5 days of the attack (133, 134).

One small randomized placebo-controlled trial of IVIg in 17 patients with autoimmune epilepsy associated with LGI or Caspr2 autoantibodies showed a significant difference in seizure reduction compared to placebo (135). In our experience, patients with LGI1 autoantibodies respond best to corticosteroids and IVMP followed by prolonged high dose oral steroids with a slow taper seems most effective. Anti-epileptic drugs are generally ineffective in controlling seizures in patients with autoimmune encephalitis. In a longitudinal follow-up of 110 patients with encephalitis and seizures associated with LGI1, NMDAR, or GABA-B autoantibodies, only 14% of patients achieved seizure freedom while using only anti-epileptic drugs vs. 53% of patients treated with immunotherapy. In the same study, carbamazepine was more effective than levetiracetam in reducing seizure frequencies in patients with LGI1 autoantibodies (136).

A multicenter observational study analyzed treatment responses and outcomes in 501 patients with NMDAR autoantibody-positive encephalitis for 2 years (137). Improvement within 4 weeks was observed in 53% of patients after tumor removal or first line immunotherapy (IVMP, IVIg, PLEX, or combinations). Among non-responders, 57% of patients received either rituximab or cyclophosphamide resulting in better outcomes than those who did not. A more recent study conducted in Western China on 244 patients with NMDAR autoantibodies followed for a median of 40 months found a relapse rate of 16%, while fatalities were observed in 7% of cases. Most patients improved with immunosuppressive treatment, and disturbances of consciousness during the first month independently predicted poor outcomes (138).

Treatment of CNS autoimmunity secondary to TNFα inhibitors or ICI should ideally require a combination of traditional acute immunotherapies (IVMP, IVIg, and/or PLEX) and withdrawal of the triggering agent. In cancer patients treated with ICI, however, the risk and benefits of ICI withdrawal should be carefully weighted based on cancer status and the severity of neurologic manifestations (121). Improvement has also been reported after immunotherapy in patients who did not discontinue ICI (126).

### Maintenance Immunotherapy

Long-term immunosuppression aims to prevent relapses after the acute attacks, or worsening of neurological manifestations in patients with a chronic progressive course associated with certain autoantibodies. The choice of the immunosuppressive agent is mostly based on the type of autoantibody detected and safety profile of the drug. Neurological CNS syndrome associated with autoantibodies targeting cell-surface proteins are generally treated with antibody-depleting or B-cell-depleting agents (e.g., rituximab) while syndromes with autoantibodies targeting intracellular antigens that are thought to be part of a predominantly cytotoxic process are preferentially treated with agents depleting all types of immune cells (e.g., azathioprine, mycophenolate mofetil, cyclophosphamide) although some overlap exists in treatment regimens. However, a reduced effectiveness of B-cell depleting agents with autoantibodies targeting intracellular antigens has not been proven and the mechanism of action of rituximab might also indirectly involve T-cells and depletion of B cells may also reduce T cell function and depleting T-cells may reduce B-cell function. Commonly used long-term immunosuppressants and related administration regimens are listed below:

*Rituximab* is an anti-CD20 monoclonal antibody. It is widely used worldwide due to the good tolerability, limited risk of adverse reactions, and fast onset to action. There are two main regimens of intravenous administration for rituximab in adults: (1) Induction with two 1 g-infusions 2 weeks apart and the same regimen every 6 months; and (2) induction with 375 mg/m^2^ of body surface area/week for 4 consecutive weeks, then periodic reinfusions with half of the initial dose (1 reinfusion/week for two consecutive weeks). The timing of reinfusions can be either fixed (generally at 6 months intervals), or guided by monthly/bimonthly monitoring of CD19-positive cell count (in this case the drug is reinfused when the proportion of CD19 positive cells increases >1%). Side effects include infusion reactions and risk of infections sometimes in the setting of a secondary hypogammaglobulinemia; thus, monitoring of total IgG levels 6 monthly can be considered.

*Azathioprine* is an antagonist of purine synthesis and consequently DNA/RNA production for the proliferation of white blood cells. It is generally administered orally with a recommended total daily dose of 2–3 mg/Kg. Azathioprine commonly takes 6–8 months to become effective so that a prolonged, concomitant taper of oral steroids is often needed in conjunction. Side effects include rash, infection, macrocytic anemia, hypersensitivity reactions, pancreatitis, elevated liver enzymes and increased risk of tumors (e.g., lymphoma and skin cancers). Testing thiopurine methyltransferase (TMPT) enzyme activity before starting is mandatory and those with deficiency are at increased risk of severe bone marrow toxicity and in this population the medication should be avoided.

*Mycophenolate Mofetil* is another oral inhibitor of purine metabolism, mainly acting on lymphocytes. The recommended starting dose in adults is 500 mg twice daily and increasing to a goal dose of 1,000 mg twice daily. This drug becomes effective in ~2–3 months. Side effects include GI disturbance, infections, and increased risk of tumors (e.g., lymphoma and skin cancers).

*Cyclophosphamide* acts by inducing cell apoptosis via induction of irreversible DNA alterations. It is generally reserved for severe cases refractory to other immunotherapies due to the strong immunosuppressive effect and greater risk of adverse events which include nausea and vomiting, alopecia, hemorrhagic cystitis, agranulocytosis, infertility and increased risk of tumors (e.g., lymphoma and skin cancers). The recommended dose in adults is 1,000 mg/m^2^ monthly intravenously or 1–2 mg/kg once daily by mouth. In general, treatment is limited to 6 months after which transition can be considered to one of the other immunosuppressants mentioned above.

Novel therapies targeting key specific proteins of the inflammatory cascade have also been proposed as promising and highly effective treatments for antibody-mediated CNS disorders and can be considered for refractory cases. These include inhibitors of certain interleukins or their receptors (e.g., IL-6), and specific complement proteins (e.g., C5) (139, 140). Hematopoietic stem cell transplantation has also been investigated (141).

A number of monoclonal antibodies have recently been proven to be highly effective for relapse prevention in patients with AQP4 autoantibodies by randomized clinical trials, including eculizumab (anti-C5 complement protein) (142), satralizumab (anti-IL-6 receptor) (143), inebilizumab (anti-CD19) (144), and the better-known rituximab (anti-CD20) (145). A full review of the dosing and side effects of these medications is beyond the scope of this paper but is reviewed elsewhere (146). Interestingly, the demyelinating disorder associated with MOG autoantibodies seems to respond less robustly to rituximab compared to what is seen with AQP4 autoantibodies (147, 148). Monthly IVIg infusions may be effective in preventing relapses with MOG autoantibodies but randomized clinical trials are needed to help guide treatment in this disease.

### Oncologic Treatments

In patients with paraneoplastic antibody-mediated CNS disorders, cancer removal should be prioritized even in patients with severe acute disability. Early cancer removal has been associated with improved outcomes and better treatment response (137). Other cancer treatments are also often utilized (chemotherapy, radiation) and depend on the cancer type. For some cancers, the recommended treatment may also act on the autoimmune CNS disorder (e.g., rituximab in the context of R-CHOP to treat lymphoma). Caution is advised in the addition of an immune-checkpoint inhibitor in a patient with a pre-existing paraneoplastic neurologic disorder as it may result in severe morbidity or mortality (126).

### Outcome and Prognosis

Outcomes are generally more favorable for patients with autoantibodies targeting cell-surface antigens, although residual cognitive impairment has been documented with many of these autoantibodies (e.g., NMDAR, LGI1) (149, 150). Decreased quality of life and school performance is not uncommon in children after encephalitis with NMDAR autoantibodies (151). The timing of immunotherapy initiation seems a main determinant of long-term functional outcome (137, 152). Autoantibodies targeting intracellular antigens rarely respond even to aggressive immunosuppression, so that stabilization of the neurologic deficit is frequently considered a good achievement. A recent study on 212 patients with GAD65 autoimmunity found complete immunotherapy response in only 1% of cases (45). In patients with paraneoplastic CNS syndromes, progression of the underlying cancer is a major cause of death (153). GFAP autoantibodies represent an exception since they generally respond well to corticosteroids (despite the intracellular location of GFAP), and rarely lead to major long-term disability (26), although outcomes can be worse among Asians (154). A minority of patients with antibody-associated encephalitis develop epilepsy after the acute phase of the disease, mostly due to chronic structural sequelae (e.g., severe atrophy of the mesial temporal pole) or antibody-mediated alteration of synaptic transmission in the long-term. The risk of secondary epilepsy seems higher with certain neural autoantibodies, including antibodies against GAD65, LGI1, and GABA_A_R (155). Management of seizures in these patients is often challenging as they might be resistant to multiple anti-epileptic drugs and immunotherapy is unlikely to be effective outside of the active inflammatory phase.

Among autoantibodies associated with CNS demyelination, those targeting AQP4 are typically associated with a worse outcome given the destructive nature of their clinical attacks which leads to a stepwise disability accumulation (156, 157). Clinical attacks associated with MOG autoantibodies are similarly severe but patients tend to recover completely or nearly completely with treatment, so that the long-term outcome in favorable for the majority of the patients, even after a highly relapsing course (158, 159). A poor outcome, however, can be observed in a minority of patients with MOG autoantibodies (<10%), often in association with relapsing brain attacks (160). Different from multiple sclerosis, a secondary progressive course is rare in patients with AQP4 and MOG autoantibodies (158, 161).

The outcome is often favorable also in patients treated with ICI who develop autoimmune CNS disorders, although severe residual disability can be observed in approximately one third of patients (126). Older age at ICI treatment initiation is a major predictor of poor outcome in these patients. ICI treatment in patients with pre-existing neurological autoimmunity may lead to irreversible neurologic worsening despite aggressive immunotherapy (126).

## Conclusions

In conclusion, antibody-mediated CNS disorders are heterogeneous with variable clinical-MRI characteristics and prognoses based on the specific underlying autoantibody. Identification of one or more neural specific autoantibodies in the serum and/or CSF of patients with a compatible clinical phenotype confirms the diagnosis but false positive results may occur especially with indiscriminate testing, which could lead to inappropriate treatment. General treatment principles exist for antibody-mediated CNS syndromes exist but most lack evidence to support their use and are often broad immunosuppressants rather than targeted treatments. Future directions for this field include developing more targeted treatments for each antibody-mediated syndrome using the success in aquaporin-4 positive NMOSD as a guide on how specific targeted treatments can be developed, proven to work and made available for patient care.

## Author Contributions

This manuscript was entirely planned and drafted by ES and EF, including drafting of the Figures and Tables. Both authors contributed to the article and approved the submitted version.

## Conflict of Interest

The authors declare that the research was conducted in the absence of any commercial or financial relationships that could be construed as a potential conflict of interest.
